# Composition and physicochemical properties of calcium silicate based sealers: A review article

**DOI:** 10.4317/jced.54103

**Published:** 2017-10-01

**Authors:** Farnaz Jafari, Sanaz Jafari

**Affiliations:** 1 Assistant Professor, Department of Endodontics, Dental School, Tabriz Branch, Islamic Azad University, Tabriz, Iran; 2 Assistant Professor, Orthodontics Department, Dentistry Faculty, Ilam University of Medical Sciences

## Abstract

**Background:**

Recently a new generation of endodontic sealers has been developed based on calcium silicate as MTA Fillapex, Endoseal MTA, Total Fill BC Sealer, EndoSequence BC Sealer, iRoot SP, Endo CPM sealer, MTA-Angelus and ProRoot Endo Sealer. A review of literature was conducted to discuss the composition, physicochemical properties, and clinical perspectives of calcium silicate based sealers.

**Material and Methods:**

A literature search was conducted in PubMed and web of knowledge databases with appropriate MeSh terms and keywords. A total of 71 studies were reviewed for data extraction.

**Results and Conclusions:**

Calcium silicate based sealers showed suitable physical properties to be used as an endodontic sealer. However, its high solubility remains an important issue. They show good performance regarding calcium ion release, film thickness, and fowability. More researches are required about features of calcium silicate based sealers before recommending them for clinical applications.

** Key words:**Calcium silicate, root canal filling materials, composition, physical properties.

## Introduction

Endodontic sealers that recently have been developed are working as sealing agents in filling root canals (1). A variety of endodontic sealers is available including zinc oxide eugenol, calcium hydroxide, glass ionomer, silicone, resin, and bioceramic based sealers (2). Bioceramic based sealers are ceramic products that are designed particularly for medical and dental applications (3). These sealers include alumina, zirconia, bioactive glass, glass ceramics, hydroxyapatite, and calcium phosphates (4). Bioceramic based sealers are categorized into two groups of calcium silicate based sealers (Mineral Trioxide Aggregate (MTA) based and non MTA based) and calcium phosphate based sealers (2). Also, another categorization of bioceramic based sealers is available in two groups of bioactive and bioinert materials due to their interaction with the close, alive tissues (5). Bioactive materials, such as glass and calcium phosphate, interact with the surrounding tissue to encourage the growth of more durable tissues (6). The physicochemical properties of sealers have been always considered because of their biological and technical importance (2).

Aiming at combining the physicochemical properties of a root canal sealer (7), a new generation of endodontic sealers has been produced based on calcium silicate as MTA Fillapex (8), EndosealMTA (9), Total Fill BC Sealer (10), EndoSequence BC Sealer (11), iRoot SP, Endo CPM sealer, MTA-Angelus and ProRoot Endo Sealer (12). Lack of an extensive review article on composition, physical properties and clinical perspectives of calcium silicate based sealers felt.

## Material and Methods

The authors searched web of knowledge and PubMed databases, using the search terms of tricalcium silicate, root canal sealers, physical properties, material characterization, mineral trioxide aggregate (MTA), calcium silicate-based sealers, calcium release and solubility with different combinations. This initial search yielded 250 articles until October 2016, 179 of which were excluded due to not consistency to the topic and language other than English or Persian. The remaining 71 met our predefined criteria and were included in this review. Two researchers independently reviewed, extracted and summarized the data. Data extraction included composition, physicochemical properties and clinical perspectives of calcium silicate based sealers (13-33).

## Results

1. Composition

 Table 1 shows the manufacturer and composition of calcium silicate based sealers.

**Table 1:**
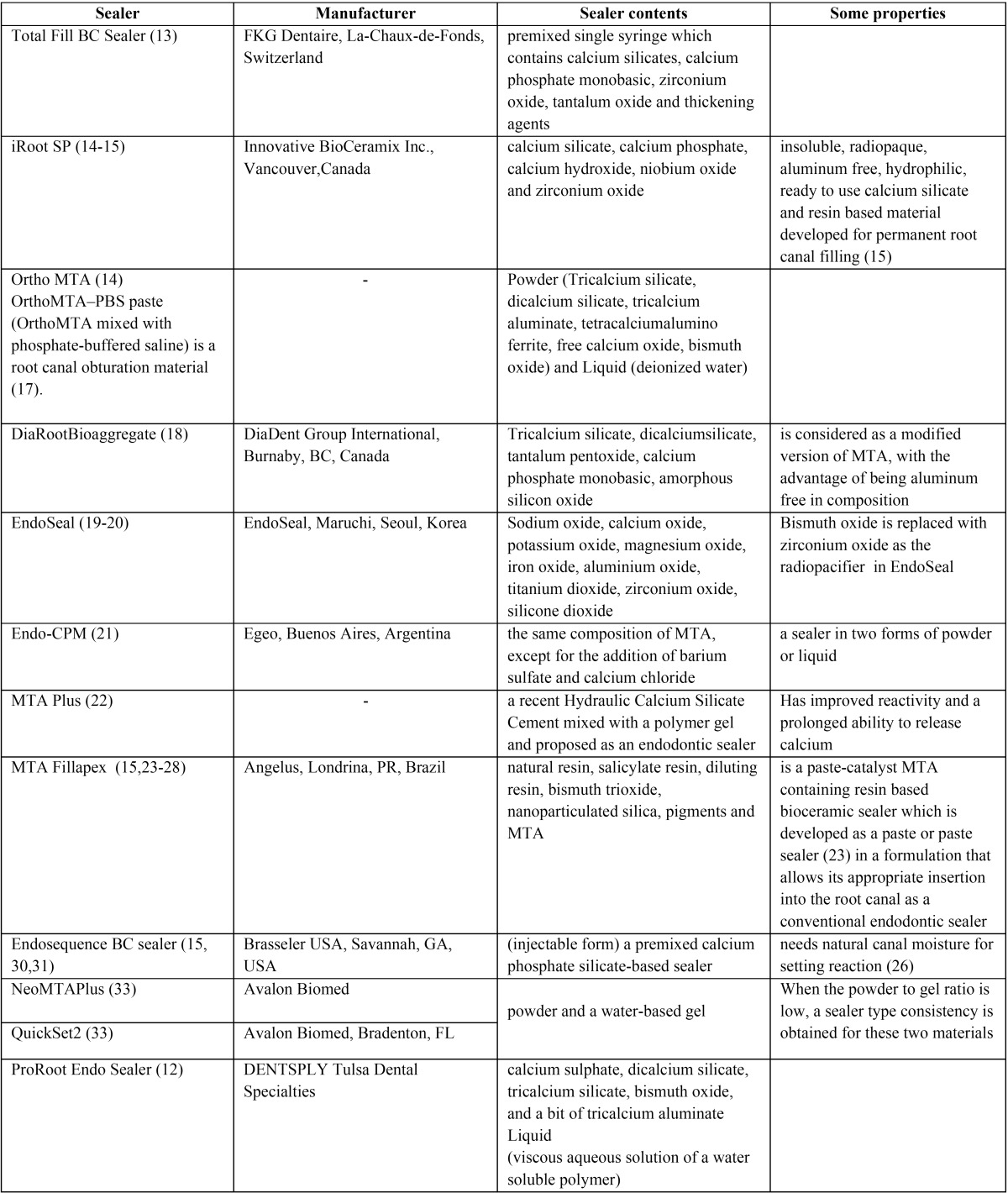
Composition of calcium silicate based sealers.

2. Physicochemical properties

-Radioopacity 

Radioopacity, a well-known characteristic of endodontic sealers, should exist in any root canal filling materials with a certain degree in order to evaluate the quality of root filling function (34). Two methods of standard discs and tissue simulator were used to evaluate radio opacity in one study which indicated that it was higher in AH Plus than MTA Fillapex and Endo CPM (34). Radioopacity of Endo CPM sealer was 6 mmAl (35). Also, radiopacity of MTA Fillapex and AH-Plus were 3.9 and 18.4 mmAl respectively (36). Xuereb (37), found the value of 10.8 and 4.3 for radioopacity of Endosequence BC and MTA Fillapex sealers. However, another study demonstrated the radioopacity of Endosequence BC sealer and AH Plus to be 3.84 and 6.90 mm Al respectively (38). The radiopacity of Endoseal was lower than AH Plus (9).

-Setting time

The ANSI/ADA Specification number 2 requires that the setting time of a sealer shall be within 10% of that stated by the manufacturers (39). The final setting time was determined to be 85.66 (±6.03) minutes for Biodentine and 228.33 (±2.88) minutes for MTA (40). Endosequence BC Sealer required at least 168 hours to reach the final setting using the Gilmore needle method, and its microhardeness significantly declined when water was included in the sealer (41). Endosequence BC sealer and MTA Fillapex could not be set in dry condition within 3 days whereas they set in contact with the physiologic solution (HBSS) (37). The setting time values for EndoSequence BC Sealer and MTA Fillapex in moist conditions were 22.3 and 19.3 minutes respectively (37). These results are in contrast with another study, finding that the setting time of these materials is 2.7 hours (42). When there are minimal amounts of fluids in contact with the materials, dry conditions are not valid *in vivo*. An equivalent of 20 cm water pressure investigating the hydraulic conductance of dentin (43).

Dentin permeability is mainly caused by the dentinal tubules present. Permeability in dentin can be reduced by the apposition of tertiary dentin, the deposition of crystalline calcium phosphate, or the presence of the smear layer and coagulation products (37).

-Solubility

Solubility is the mass loss of a material during a period of immersion in water. The solubility of a sealer should not be superior to three percent by mass based on specification number 57 of ANSI/ADA (44). The solubility of both iRoot SP and MTA-Fillapex was high (20.64% and 14.89%, respectively), which are not fulfill ANSI/ADA requirements (45,46). Amoroso-Silva (47) showed MTA Fillapex sealer possess a higher solubility and quantity of gaps in the dentin/ sealer interface when compared to the AH Plus sealer. Nevertheless, in another study, the solubility of MTA-Fillapex (48) and EndoSequence BC (42), were in line with ISO 6876/2001. MTA-Angelus also has low solubility (46) because of an insoluble matrix of crystalline silica within itself that preserve its integrity even in the presence of water (49). The solubility and water absorption increased significantly over time for both MTA Fillapex and AH Plus in 1 to 28 day period (48). MTA Fillapex had a higher solubility that guttaflow (50). MTA Fillapex and Endosequence BC sealers have a higher solubility than AH Plus (42). Solubility and disintegration of MTA Fillapex are lower than AH Plus (48). The solubility of AH Plus and MTA Angelus agreed with ANSI/ADA’s requirements, whereas iRoot SP, MTA Fillapex, and Sealapex were unlike ANSI/ADA’s protocols (46). Morphological changes in both outer and inner surfaces after the solubility test were observed in SEM/EDX analysis of all sealers (46). Similar solubility, but a higher dimensional change of Endoseal was observed between Endoseal and AH Plus (9).

-Calcium release 

For calcium ion release test, mostly, high release of calcium was indicated in the sealers in three hours assessment and by decreasing values over time (51). In contrast to AH Plus, iRoot SP, MTA Fillapex sealers showed high levels of Ca (2+) ion release (46).

Calcium release of MTA Fillapex was superior than guttaflow (50) and inferior than iRoot SP (52). EndoSequence BC Sealer had high tendency to release calcium ions than AH plus sealer (38). The highest leaching of calcium ion was exhibited by EndoSequence BC Sealer followed by MTA Fillapex (37). Phosphorus was leached in solution in higher quantities in EndoSequence BC Sealer compared with MTA Fillapex, which exhibited a negative value, indicating that there was the uptake of phosphorus from the solution rather than leaching (37).

-pH, alkalinizing activity and correlation with antibacterial properties

Zhang *et al.* (53) tested the antibacterial activity of iRoot SP sealer *in vitro* against *E. faecalis* found that iRoot SP showed a pH value of 11.5 even after setting, but its antibacterial effect was greatly diminished after seven days. EndoSequence BC Sealer has also been shown to have high pH (>11) (38). iRoot SP showed more pH value than MTA Fillapex (52).

The pH value of the Endo CPM was higher than that of MTA Fillapex (>11); however, the bacterial inhibition zone produced by MTA Fillapex was greater than that produced by Endo CPM (54). Antibacterial activity of MTA Fillapex was related to the existence of resin as the main factor (54). However, the ability to maintain antibacterial activity after setting even with their primary high pH was observed in any of the sealers (54). The value of pH was slightly higher for MTA-Angelus than ProRoot (55).

MTA Fillapex has higher alkalinity and pH than AH Plus (36,56) and higher alkalinizing activity than guttaflow (50). MTA Fi-llapex sealer had the lowest pH at 3 and 24 hours approximately 7.5 and increasing PH values over time (51). Higher alkalinity, was observed in Endoseal when compared to AH Plus (9). EndoSequence BC Sealer pH values greater than AH Plus sealer (38).

Dudeja stated that initial dressing of calcium hydroxide followed by obturation with Gutta-percha and iRootSP and MTA Fillapex sealers may be considered as an alternative treatment modality for inflammatory resorption and it is beneficial when compared to the long-term calcium hydroxide application (52). The reason for this phenomenon is calcium ion release performed by bioceramic sealers.

Also, MTA based sealer demonstrate higher PH values than resin based sealers, MTA, calcium enriched mixture (CEM) cement, and Portland cement generated more alkalinity and the pH of 9.47-10.80 (57).

-Flowability, film thickness and dimensional stability

Flow determines the ability of sealers for filling the irregularities, and it is the viscosity that determines the flow characteristics (58). According to ISO 6786/2001, a root canal sealer should have a flow rate of not less than 20mm (12). MTA Fillapex had flowability higher than 20 mm and a film thickness lower than 50 µm (51). MTA Fillapex was significantly more flowable than AH Plus by the results of Zhou H *et al.* (42) and Silva EJ *et al.* (56). Others stated similar flow, film thickness and inferior compressive strength of MTA Fillapex when compared to AH Plus sealers (45). Flow of Endoseal was higher than AH Plus (9). Also, they were a similarity between MTA Fillapex and Endosequence BC sealers (42). Film thickness of MTA Fillapex is higher than both AH Plus and Endosequence BC sealer (42).

Endosequence BC Sealer (38) showed flow according to ISO 6876/2001 recommendations. The flow test revealed that BC Sealer and AH Plus presented flow of 26.96 mm and 21.17 mm respectively (38).

3. An Introduction to X-Ray Diffraction, Elemental microanalysis, and micromorphology techniques 

To investigate most crystalline products in a cement sample, X-ray diffraction is used as an efficient method to study cement structures (59). Different items such as interspersed granules with approximate 1–3 µm wide, and C (from salicylate and natural resins), O, Si, Al (4.93 wt%), S, Ca (5.37 wt%) and Cl (all from calcium silicate component, which is MTA), Ti (from pigments), and Bi (from radiopacifier) were displayed in a uniform surface of Freshly mixed MTA Fillapex. It is mention worthy that EDX showed a reduction in level of the constitutive elements like Si (1.11 wt%), Ti (1.19 wt%) and the disappearance of S and Bi fo-llowing soaking in HBSS. Moreover, the sharp enhancement of Ca (20.18 wt%) and the appearance of P (13.04 wt%) and elements from HBSS was also observed. Globular precipitates in the size of 2-8µm wide were used in order to cover the surface area. Besides, the agglomerates were with uncoated zones and the matrix was particular with the following characteristics.

The ratio of Ca/P for the whole picture area whether coated or uncoated zones was 1.2. More investigations revealed a non-homogeneous potential for nucleating Ca-poor non apatitic calcium phosphates for this substance (50). After immersing the mentioned MTA Fillapex sealer in HBSS, it was observed that porous matrix is interspersed with cement and bismuth-rich particles. The presence of Tricalcium silicate as well as bismuth oxide and silicon oxide was proven by XRD analyses. No hydration was found on particles of cement. Moreover, XRD analyses did not show any calcium hydroxide peak (37).

In the following, a highly dense matrix which had the lowest porosity was shown by the set EndoSequence BC Sealer (37) immersed in HBSS. The particles of cement were used to intersperse rich-zirconium particles (with a composition based on calcium and silicon elements). Moreover, the rectangular-shaped particles which were rich in phosphor and calcium were found. Some peaks were detected through phase analysis for materials such as tricalcium silicate, calcium phosphate, zirconium oxide, and calcium hydroxide. iRoot SP, and MTA Fillapex sealers proved high levels of calcium and carbon on the contrary to AH Plus when they were evaluated with calcium and carbon (46).

The compact and generally homogeneous external surfaces of iRoot SP with some porosities were proven by Borges (46). For iRoot SP, O, Ca, Zr, C and Si peaks were shown through carrying out an EDX analysis of the area in a decreasing order. Accordingly; MTA Fillapex showed a surface mostly compact and homogeneous, when EDX, in decreasing order, exhibited C, Zr, O, W, Ca and Si peaks.

4. Effect of material composition on physicochemical properties 

New generation calcium silicate based sealers: Endosequence BC sealer use pure tricalcium silicate to avoid the aluminate phase and also any heavy metal inclusions that are synonymous with the use of Portland cement (60).

Radioopacifiers: The radiopacifier particle size had limited effect on the sealer microstructure and chemical properties (36). However, the use of barium zirconate as radiopacifier further intensified the release of calcium hydroxide compared with those using zirconiumoxide (60). Calcium phosphate was also deposited on the material surface in the light-curable resin modified materials when barium zirconate was used as radiopacifier (60). The materials that used barium zirconate as radiopacifier exhibited leaching of barium in solution, particularly the water based materials (60). The clinical implications of barium leaching from dental materials need to be investigated further.

Resin: Although there was no evidence of hydration in the light-curable resins like Bis-GMA/TEGDMA, calcium hydroxide was leached in solution (60).

## Conclusions

Composition: When concerning the composition of MTA-based sealers, iRoot SP, DiaRoot Bioaggregate and Endosequence BC sealer are aluminate free calcium silicate based sealers. However, OrthoMTA, EndoSeal, EndoCPM, MTA Plus and ProRoot endo sealer contains it. The presence of aluminium compounds is a principal disadvantage of Portland Cement derived materials, because of calcium sulfate aluminates which release aluminium ions into biologic systems (61). Aluminium is toxic to osteoblasts and inhibits bone mineralization. It also induces renal dystrophy, dementia, Alzheimer and toxic effect on red blood cells, parathyroid glands and chromosomes (62,63).

Radioopacifiers suitable for dental applications include barium sulfate, zirconium oxide, bismuth oxide, tantalum oxide and mixtures of them. Radioopacifier for Endoseal and iRootSP is zirconium oxide, while for OrthoMTA and ProRoot endo sealer is bismuth oxide and radoopacifying agent for EndoCPM is barium sulfate.

The effects of bismuth oxide concentration in the biological and physicochemical properties of MTA have been questioned (64-66). Previous studies have demonstrated interferences with cell viability and growth (65,67), with the hydration mechanisms of MTA (68) and negative effects on the compressive strength of the cement (69). Another problem is discoloration of bismuth oxide containing sealers in contact with sodium hypochlorite (70).

Radioopacity: The results of radioopacity of different MTA-based sealers showed their lower radioopacity in comparison with famous resin based sealers. However, it is not an important issue because of application of sealers together with gutta-percha. The radioopacity value of selears from the most to least value is as the following: AH Plus, Endosequence BC, Endo CPM and MTA Fillapex.

Setting time: There was limited data for setting time of MTA-Based sealers. Taking together the data for different databases, it is important to simulate 20cm H2O hydraulic pressure of dentine for evaluating setting time of sealers. When assessing their criteria, both Endosequence BC and MRA Fillapex showed acceptable setting time. However, in the dry condition setting time values for them in high and not acceptable.

Solubility: Thorough screening of results of articles on the solubility of MTA-based sealers it can be concluded that most of the sealers of this type show high solubility and dimensional and structural change after immersion in water when comparing with the standard resin based sealers. So, clinical application of them should be conducted with caution, especially in open apex teeth.

Calcium ion release: Ca ion release in iRootSP and Endosequence BC is more than MTA Fillapex and in MTA Fillapex is more than AH Plus sealer.

PH and alkalinity: Overall alkalinity of tested calcium silicate based sealers are more than AH Plus. The most PH value was observed for iRootSP, EndoCPM and EEndosequence BC sealer followed by MTA Fillapex and EndoSeal.

Flow: Overall flowability of tested calcium silicate based sealers are more than AH Plus. The value is similar between MTA Fi-llapex, Endosequence BC, and EndoSeal

XRD: MTA Fillapex sealer uptakes phosphorus and carbon from tissue fluids. The set in contact with tissue fluid form is porous and does not contain calcium hydroxide peak in XRD. While set Endosequence BC in contact with tissue fluid is dense and rich for calcium phosphate and calcium hydroxide. iRootSP contains homogeneous surface with low porosity, which contains calcium and oxygen as the main component. However, main component for set in tissue fluid MTA Fillapex is calcium and phosphorus.

Calcium silicate based sealers showed suitable physical properties to be used as an endodontic sealer. However, its high solubility remains an important issue. Resin based sealers show better performance in radioopacity, although calcium ion release, film thickness and fowability of calcium silicate based sealers were better. Further studies in different settings about other physico-chemical features of calcium silicate based sealers are needed before recommending them for clinical applications.
